# Macula Densa Nitric Oxide Synthase-1 Controls Renin Release and Renin-Dependent Blood Pressure Changes

**DOI:** 10.24976/Discov.Med.202335177.53

**Published:** 2023-08

**Authors:** Catherine Liu, Ximing Wang, Colby Parris, Qi Pang, Muhammad Usman Naeem, Lei Wang

**Affiliations:** 1 Department of Molecular Pharmacology and Physiology, University of South Florida College of Medicine, Tampa, FL 33620, USA; 2 Department of Neurosurgery, Wayne State University School of Medicine, Detroit, MI 48202, USA; 3 Hypertension and Kidney Research Center, Morsani College of Medicine, University of South Florida, Tampa, FL 33620, USA

**Keywords:** macula densa nitric oxide synthase1, renin release, hemorrhagic shock

## Abstract

**Background:**

The function of macula densa nitric oxide synthase 1 (NOS1) in the regulation of renin release is controversial. This study was conducted to further elucidate the role of macula densa NOS1 in renin release and blood pressure regulation in response to salt challenges and hemorrhagic shock.

**Methods:**

To investigate the specific role of NOS1 in the macula densa within the kidney in response to varying sodium concentrations in the diet, tissue specific macula densa NOS1 knockout (MDNOS1KO) and wild type (WT) mice were subjected to sequential low (0.1% NaCl) and high (1.4% NaCl) sodium diets. Separate groups of mice, consisting of both MDNOS1KO subgroup and WT subgroup, were induced hemorrhagic shock by retro-orbital bleeding of 12 mL blood/kg body weight. Mean arterial pressure (MAP) was measured by a radio-telemetry system. Plasma renin concentration (PRC) was measured with the radioimmunoassay for both sodium diet and hemorrhagic shock experiments.

**Results:**

PRCs were 371 ± 95 and 411 ± 68 ng/mL/hr in WT and MDNOS1KO mice fed a normal sodium diet, respectively. Low salt intake stimulated an increase in the renin release by about 260% in WT mice (PRC = 1364 ± 217 ng/mL/hr, *p* < 0.0001) compared to the PRC under normal salt diet. However, the stimulation was significantly blunted in MDNOS1KO mice (PRC = 678 ± 104 ng/mL/hr, *p* < 0.001). High salt intake suppressed the PRC to about 61% of the PRC level under a normal salt diet (*p* < 0.0001). Deletion of macula densa-NOS1 further inhibited renin release to 33% of the levels of a normal salt diet. Hemorrhagic shock induced about a 3-fold increase in PRC in WT mice, but only about a 54% increase in the MDNOS1KO mice (*p* < 0.0001). The MAP values were substantially greater in WT mice than in MDNOS1KO mice within the first 6 hours following hemorrhagic shock (*p* < 0.001). Thus, WT mice showed a much quicker recovery in MAP than MDNOS1KO mice.

**Conclusions:**

Our study demonstrated that macula densa NOS1 plays an important role in mediating renin release. This mechanism is essential in maintaining blood pressure under hypovolemic situations such as hemorrhagic shock.

## Introduction

Renin-angiotensin-aldosterone system (RAAS) plays a fundamental role in the regulation of blood pressure. In this hormonal cascade system, renin is the rate-limiting step in the production of angiotensin II. Multiple factors, including nitric oxide (NO) produced by the macula densa cells, are involved in the control of renin release from juxtaglomerular cells [(Santos, Oudit et al. 2019), 2].

Nitric oxide synthase 1 (NOS1) is the main NOS isoform produced in the macula densa cells [2,3]. Although the role of macula densa-produced NO in renin release has been extensively investigated for decades, the results are not consistent. Many investigators have reported a stimulatory function of NO in renin secretion [4, 5], while others have reported no influence or an inhibitory effect of NO on renin secretion [6, 7]. In addition, the functional significance of the macula densa NOS1 in the control of renin-dependent blood pressure changes has not been investigated. There are three splice variants of NOS1 α-, β-, and γ expressed in macula densa cells, where NOS1β is the main splice variant and responsible for the majority of NO generation by macula densa [8, 9]. To completely delete all three splice variants of NOS1 at the macula densa, a macula densa-specific NOS1 knockout (MDNOS1KO) mice were produced by crossing Na-K-2Cl cotransporter (NKCC2)-Cre with NOS1flox/flox mice (NKCC2^Cre^/NOS1^tm2Plhflox^) [9]. Since the expression of NOS1 is very little in the thick ascending limb of the loop of henle compared with that in the macula densa cells [10, 11], the MDNOS1KO strain is actually reflected a macula densa–selective NOS1KO model. The removal of NOS1 in the macula densa has been verified by immunofluorescent staining of the NOS1 protein and NO generation measurement in isolated perfused macula densa from MD-NOS1KO and littermates of C57BL/6 (wild type (WT)) [9].

In the present study, we took advantage of these MDNOS1 KO mice to further evaluate the contribution of the macula densa-NOS1-derived NO in the renin release *in vivo*. The plasma renin concentration (PRC) in response to salt challenges was measured in WT and MDNOS1KO mice. In addition, to examine the functional significance of macula densa-NOS1 in renin-dependent blood pressure changes, we induced hemorrhagic shock in conscious WT and MDNOS1KO mice. PRC and mean arterial pressure (MAP) were measured.

## Methods

### Animals

All animal use and welfare adhered to the National Institutes of Health Guide for the Care and Use of Laboratory Animals. All animal experiments were performed at Comparative Medicine animal facility at the University of South Florida following protocols approved by the Institutional Animal Care and Use Committee (IACUC, IS000011615R) at the University of South Florida.

The macula densa-NOS1 KO (NKCC2^Cre^/NOS1^tm2Plhflox^, MDNOS1KO) mice with a C57BL/6 background and the littermate NOS1^flox/−^ control mice were bred at the University of South Florida. Male MDNOS1KO and control (10–12 weeks of age) mice were used in this study [9]. The animals were given normal (0.4% NaCl), low (0.04% NaCl), and high salt diets (1.4% NaCl: normal salt diet plus 1% NaCl drinking water) (2018 Teklad Global 18% Protein Rodent Diet, Envigo, Haslett, MI, USA) for 7 days on each diet. The other electrolyte levels and energy content were the same in the diets. All parameters were first measured on a normal sodium diet, followed by a low sodium diet, and then a high sodium diet in the same group of animals. Thirteen mice were used for this experiment (n = 7 for littermate NOS1^flox/−^ control mice/WT mice and n = 6 for MDNOS1KO mice). Experiments for hemorrhagic shock and MAP measurement were performed in separate groups of mice. Twenty mice were used for the hemorrhagic shock experiment (n = 10 for littermate NOS1^flox/−^ control mice/WT mice and n = 10 for MDNOS1KO mice). At the end of experiment, the mice were euthanized by cervical dislocation under anesthesia after collection of samples. Death of mice was verified prior to carcass disposal following the instruction of the IACUC at University of South Florida. This method is consistent with the recommendations of the Panel on Euthanasia of the American Veterinary Medical Association. All chemicals were purchased from Sigma (St. Louis, MO, USA) except as indicated.

### Glomerular Filtration Rate (GFR) Measurement in Conscious Mice

Glomerular filtration rate (GFR) was measured in conscious mice. Following the retro-orbital bolus injection of fluorescein-isothiocyanate (FITC)-sinistrin (5.6 mg/100 g body weight, Fresenius Kabi, Linz, Austria) and series plasma sample collection, a plate reader (Cytation5, BioTek, VT, USA) was used to measure the fluorescent intensities of FITC-sinistrin in the plasma samples as described previously [12, 40]. The two-compartment model of two-phase exponential decay (GraphPad Prism9, San Diego, CA, USA) was applied to calculate the GFR values. GFR was presented as microliters per minute (μL·min^−1^).

### Mean Arterial Pressure (MAP) Measurement with a Telemetry System

Telemetry transmitters (PA-C10, DSI, St Paul, MN, USA) were implanted as we described previously [13]. After 10 days of recovery from surgery, the MAP of the mice was measured for 30 seconds every 10 minutes each day for 5 days as baseline. The MAP and heart rates were recorded continuously for the first 48 hours after hemorrhagic shock.

### Hemorrhagic Shock Induction

Basal MAP was measured for 5 days. Hemorrhagic shock was induced as we previously described [14,15]. Briefly, mice were anesthetized with isoflurane as described above and 12 mL blood/kg body weight was withdrawn within 3 min using a standard heparinized micro-hematocrit capillary tube through the right retro-orbital sinus with the body temperature controlled in the range of 36.8–37.0 °C. The animals were kept on a heating pad until fully conscious before being returned to their housing cages. The sham groups for both WT and MDNOS1KO mice underwent the same anesthesia procedure as the hemorrhagic shock groups, albeit without any bleeding.

### Plasma Renin Concentration (PRC) Measurement

PRC measurement was performed on the 7th day of normal salt (NS), low salt (LS), or high salt (HS) diets. For hemorrhagic shock experiments, PRC was measured at 0, 1, 3, 9, 15 and 20 hours post hemorrhagic shock as previously reported [16]. The mice were lightly anesthetized with 1.5% isoflurane. Blood samples (~25 μL/sample) were collected from the retro-orbital plexus and centrifuged for 10 minutes at 1500 × g. Plasma samples (~10 μL) were obtained and incubated with excess porcine angiotensinogen (0.4 μmol/L; Sigma, St. Louis, MO, USA) for 20 min. The amount of angiotensin (Ang) I generated after incubation was used to calculate the PRC with the Ang I enzyme immunoassay (EIA) kit (Bachem, San Carlos, CA, USA). PRC was expressed as the amount of Ang I generated per hour per milliliter of plasma.

### Plasma Aldosterone Concentration (PAC) Measurement

Plasma aldosterone concentration (PAC) was measured at the end of different sodium diets with a standard Radioimmunoassay (RIA) kit (COAT-A-COUNT Aldosterone, Products Corporation, Los Angeles, CA, USA) as described previously [17, 18].

### Immunofluorescence staining

Paraffin embedded Kidney samples for both WT and MDNOS1KO mice were sectioned into 4-μm slices. Immunofluorescence staining of the kidney slices with the rabbit anti-Renin primary antibody (Abcam, AB212197, Waltham, MA, USA) and a fluorophore-conjugated secondary antibody Donkey Anti-Rabbit IgG H&L (Abcam, ab150075, Waltham, MA, USA) was completed as described previously [1,2]. The tissue sections were photographed with a fluorescence microscope (Keyence BZ-X710, Itasca, IL, USA) and analyzed with Fiji/ImageJ. Five images per kidney were acquired and the relative renin density was calculated by dividing the renin-positively stained area by the kidney area. The morphometric analyses were conducted in a blind manner with respect to the experimental procedures.

### Renin mRNA expression measurement by Real-time PCR

To evaluate the impact of MD-specific NOS1 deletion on intrarenal RAS, we utilized Real-time PCR to measure the mRNA expression of renin, following the established methodology described earlier [3,4]. β-actin served as the reference gene for internal standardization purposes. Relative quantitative expression of renin was determined using SYBR Green I (Invitrogen Molecular Probes, Eugene, OR, USA) in a CFX Connect system (BioRad, Hercules, CA, USA). Ren1 mRNA (primer sequence: forward, 5’-ACAGTATCCCAACAGGAGAGACAAG-3’, reverse, 5’-GCACCCAGGACCCAGACA-3’) expression was adjusted with β-actin expression, and the relative changes in expression were calculated using the ΔΔCt method and expressed as relative expression in comparison to WT mice.

### Statistics

All values are expressed as mean ± SD. The comparisons of PRC, PAC and GFR responses to the changes of sodium intake and the MAP and renin concentration changes in the response to the hemorrhagic shock between the two genotypes were performed using 2-way Analysis of Variance (ANOVA (mixed)) followed by Tukey multiple comparison test with repeated measures. Statistical analysis was performed with GraphPad Prism 9. A *p*-value of < 0.05 was considered to be statistically significant.

## Results

### PRC Changes in Response to Salt Challenges in MDNOS1KO and WT Mice

**Fig. 1.**
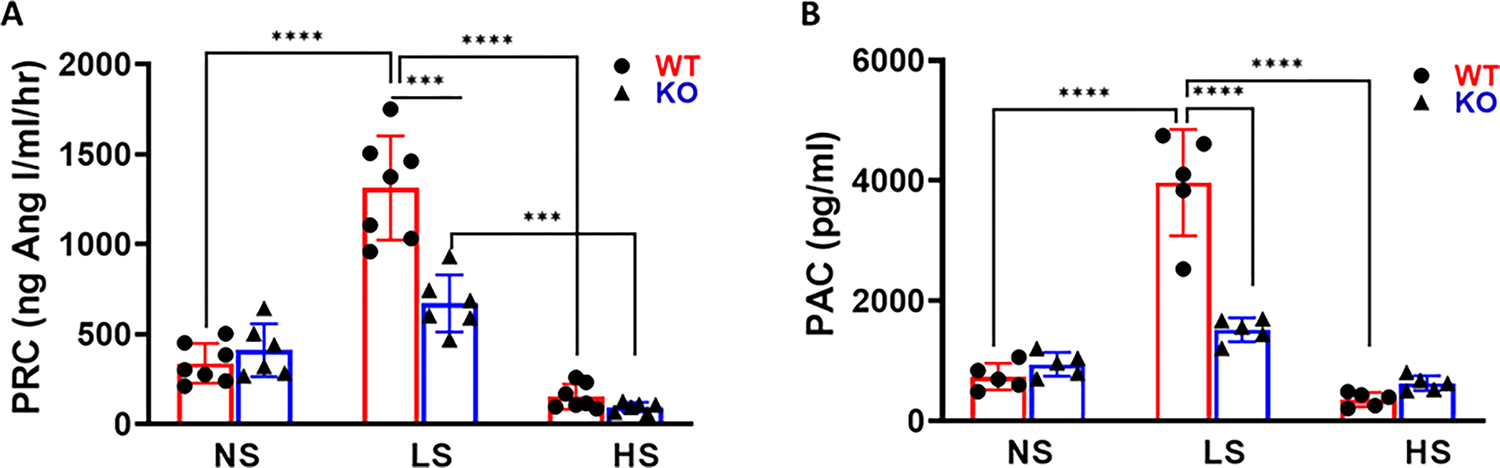
Plasma renin and aldosterone concentration response to dietary salt intake. Plasma renin concentration (PRC) (A) and plasma aldosterone concentration (PAC) (B) were measured at the end of each dietary salt intake. Restriction in sodium intake is associated with markedly elevated plasma renin concentration (PRC) (****p < 0.0001, ***p < 0.001, n = 6–7) and PAC in wild type (WT) mice but not in macula densa-NOS1 knockout (MDNOS1KO) mice (****p < 0.0001, ****p < 0.0001, n = 5). A high salt intake induced decreased PRC in both WT mice and MDNOS1KO mice (****p < 0.0001, ***p < 0.001, n = 5–7). NS, normal salt; LS, low salt; HS, high salt.

The PRC were 371 ± 95 and 411 ± 68 ng/mL/hr in WT and MDNOS1KO mice fed a normal sodium diet, respectively. These values are constant with the comparable levels of renin mRNA between WT and MDNOS1KO mice, although slightly higher levels of renin were found in WT than in MDNOS1KO kidneys based on immunofluorescence staining (Fig S1 and 2). Following the switch from a normal salt diet to a low salt diet, PRC was increased by about 260% in WT mice (*p* < 0.0001, low salt vs. normal salt; n = 6; Fig. 1A). This stimulation by the lower sodium diet was drastically blunted in MDNOS1KO mice with the PRC of 678 ± 104 ng/mL/hr. Thus, under low sodium conditions, MDNOS1KO mice had a significantly lower PRC than WT mice (*p* < 0.001, MDNOS1KO vs. WT; n = 6–7; Fig. 1A). After changing to a high sodium diet, PRC in WT was significantly suppressed to the level of about 61% that of a normal salt diet. Deletion of macula densa-NOS1 further inhibited PRC to 33% of the levels under normal salt diet.

Changes in plasma aldosterone followed a similar pattern as the PRC for both WT and MDNOS1KO mice as the salt in the diet was decreased (Fig. 1B). As the dietary sodium content increased, WT mice had significantly reduced PAC (*p* < 0.0001, low salt vs. normal salt and high salt; Fig. 1B, n = 5). However, there was no further significant suppression of PAC in the MDNOS1KO mice with the high salt diet (Fig. 1B).

### GFR of Conscious Mice under Different Sodium Diets

**Fig. 2.**
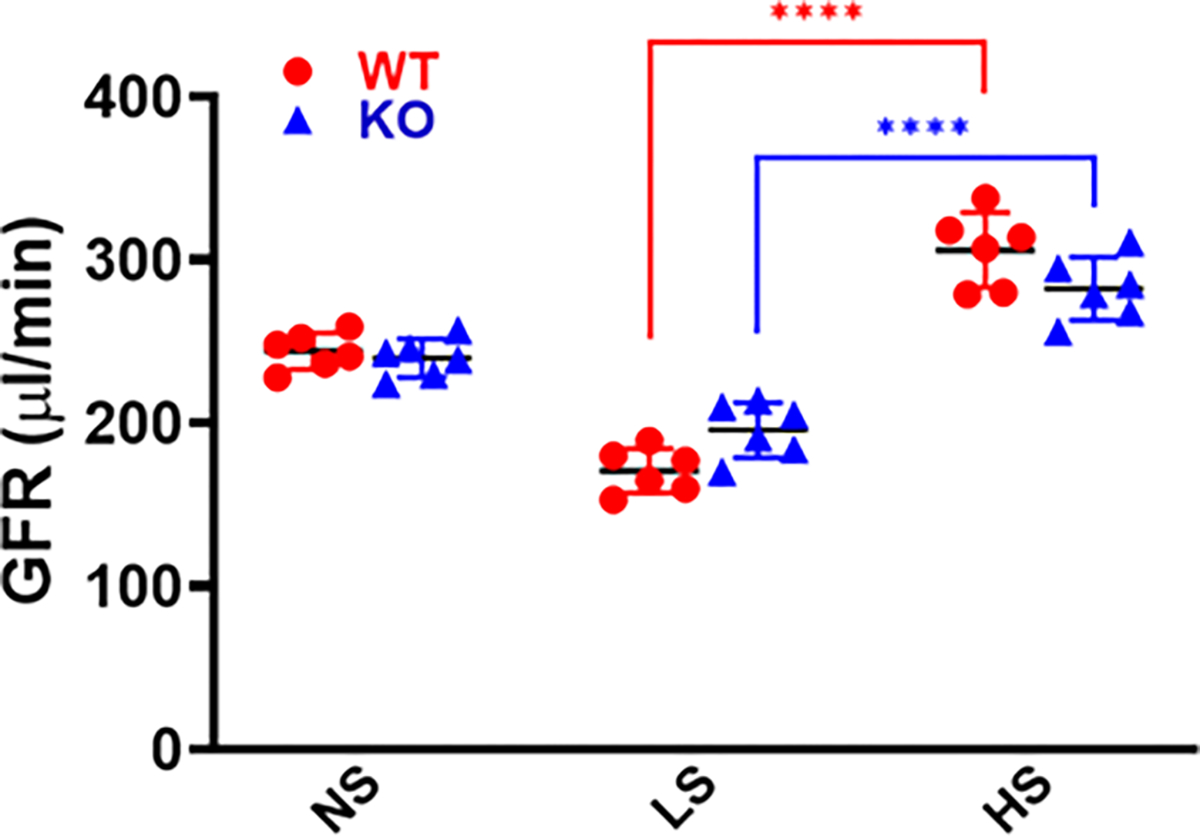
Glomerular filtration rate (GFR) response to various dietary salt intake. GFRs in conscious WT and MDNOS1KO mice were measured at one day before the end of each dietary salt intake with a single bolus intravenous injection of fluorescein-isothiocyanate (FITC)-sinistrin (****p < 0.0001, n = 6). NS, normal salt; LS, low salt; HS, high salt.

The GFRs were measured in all groups of mice one day before the end of each sodium diet intake. There were no significant differences in the GFRs between WT and MDNOS1KO mice fed a normal salt diet. The GFR reduced about 36% and 30% in WT and MDNOS1KO mice fed a low salt diet compared to normal salt diet, respectively. Changing to a high salt diet significantly increased the GFR by about 67% and 59% in WT and MDNOS1KO mice, respectively, compared to low salt diet (*p* < 0.0001, high salt vs. low salt; n = 6; Fig. 2).

### MAP and Heart Rate in Response to Hemorrhagic Shock

**Fig. 3.**
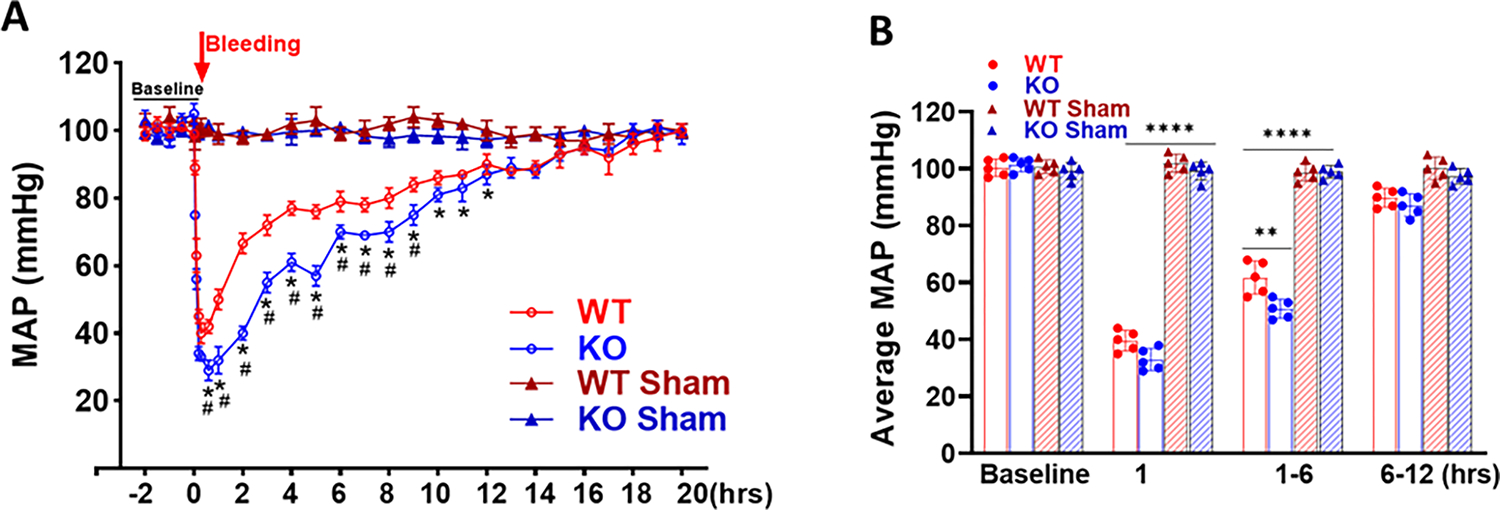
Mean arterial pressure (MAP) response to hemorrhagic shock. (A) MAP decreased to about 39 and 31 mmHg after hemorrhagic shock for WT and MDNOS1KO mice, respectively, and returned to baseline within 15 hours. Wild type mice showed quicker recovery in MAP than MDNOS1KO mice (*p < 0.0001 vs. sham; #*p* < 0.001, MDNOS1KO vs. WT; n = 5). (B) Shows the average MAP values measured in the early phage, partial and total recovery after hemorrhagic shock (****p < 0.0001, **p < 0.01, n = 5).

Basal MAP levels were similar between the WT and MDNOS1KO mice. Following withdrawal of 0.4 mL blood, the MAP quickly decreased and reached nadirs within about 30 minutes after bleeding (*p* < 0.0001, MDNOS1KO or WT vs. their shams; n = 5; Fig. 3). The MAP of the MDNOS1KO mice was significantly lower within 6 hours following hemorrhagic shock than that of the WT mice. The maximal difference was 8 mmHg (31 ± 3 vs. 39 ± 4 mmHg) (*p* < 0.01, MDNOS1KO vs. WT; n = 5; Fig. 3A,B). MAP was then gradually increased and recovered to baseline (≈98 mmHg) in about 15 hours after hemorrhagic shock (n = 5, Fig. 3). The MDNOS1KO mice exhibited delayed restoration of blood pressure compared to the WT mice. Please note that the blood pressure during the blood collection was not recorded due to anesthesia. The blood pressure should drop during anesthesia, however which recovered very quickly within two minutes as the anesthesia was slightly.

**Fig. 4.**
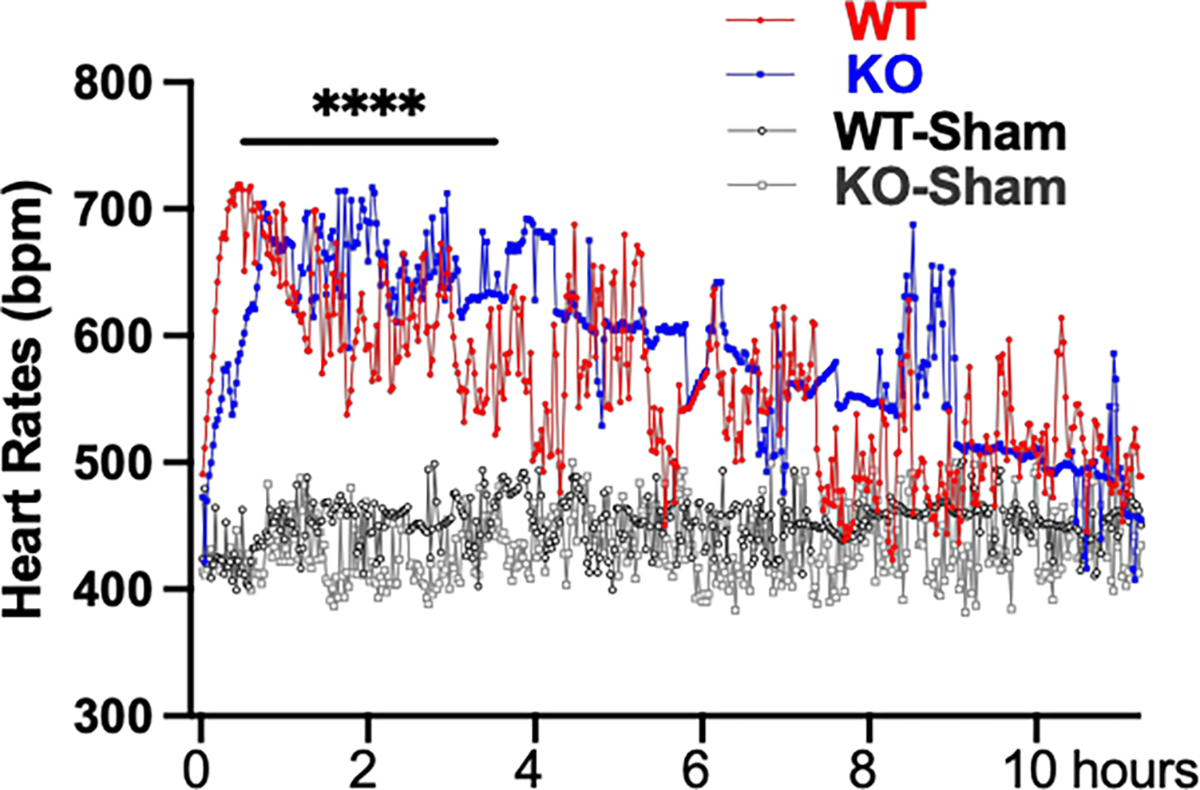
Heart rate (HR) response to hemorrhagic shock. The HRs increased about 300 bpm after hemorrhagic shock for both WT and MDNOS1KO mice and returned to baseline within 10 hours (****p < 0.0001, n = 5).

The heart rates were also monitored with radiotelemetry. There were no significant differences in basal heart rates between the two genotypes of animals. Hemorrhagic shock induced the tachycardia with the maximum increase of about 300 bpm in heart rates for both WT and MDNOS1KO mice (*p* < 0.0001, MDNOS1KO and WT vs. sham, n = 5, Fig. 4). There was a noticeable delay in the rise of the heart rates of the MDNOS1KO mice compared to the WT mice in the first hour after bleeding. However, there were not significant differences in the heart rates between the two genotypes thereafter during the measurement phase. The heart rates returned to the baseline after about 10 hours following bleeding in both WT and MDNOS1KO mice.

### PRC in Response to Hemorrhagic Shock

**Fig. 5.**
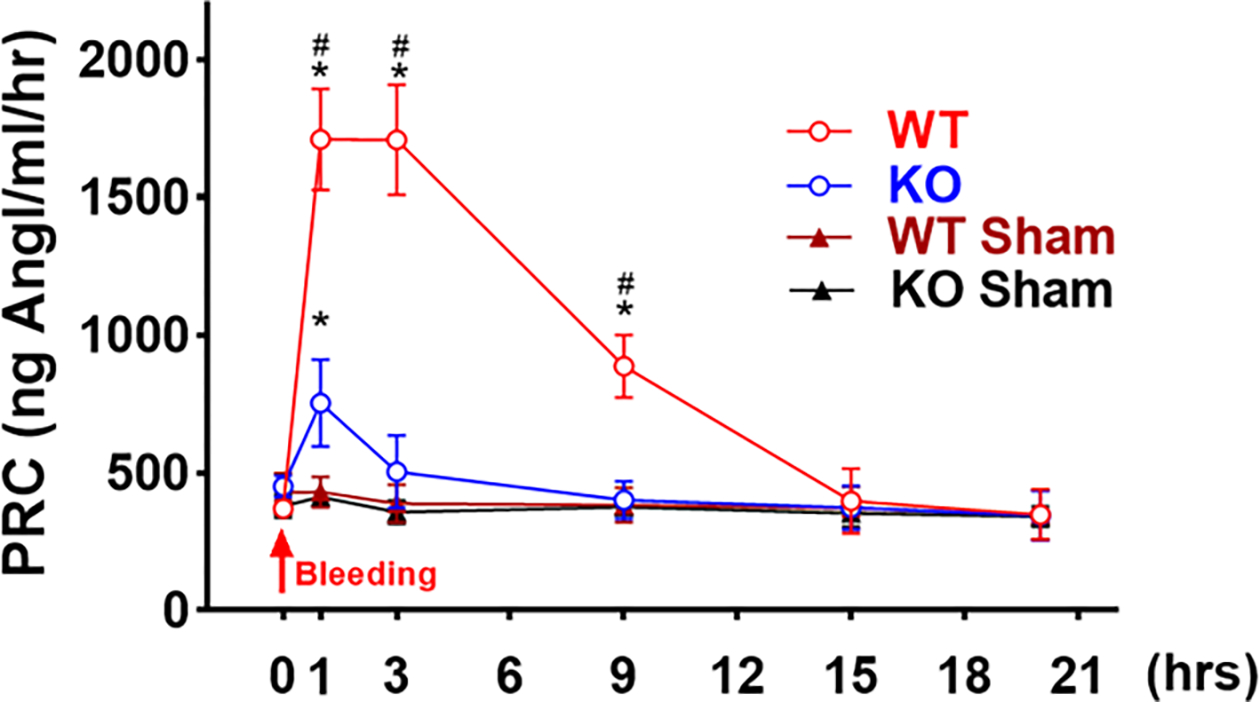
Plasma renin concentration (PRC) response to hemorrhagic shock. Plasma renin concentration (PRC) was measured in all mice at 1, 3, 9, 15 and 20 hours after hemorrhagic shock by radioimmunoassay. Hemorrhagic shock dramatically stimulated the renin release in WT mice, however, there was only a mild increase in PRC in MDNOS1KO mice (*p < 0.0001 WT or MDNOS1KO vs. their respective sham; #*p* < 0.0001 WT vs. MDNOS1KO; n = 5).

The PRC was measured at 1, 3, 9, 15 and 20 hours after hemorrhagic shock. In WT mice, PRC increased about 3-fold within the first hour and remained at a high level until 3 hours. The PRC returned to baseline within 15 hours after bleeding (*p* < 0.0001, 1 or 3 hours vs. baseline; n = 5, Fig. 5). However, there was only about a 54% elevation in PRC in the MDNOS1KO mice 1 hour after hemorrhagic shock and the PRC quickly returned to baseline after 3 hours (*p* < 0.0001, MDNOS1KO vs. WT; n = 5, Fig. 5).

## Discussion

In the present study, we directly demonstrated that lack of NO product at the macula densa inhibited the increase in PRC stimulated by a salt restriction. More importantly, for the first time, we established the functional significance of the macula densa NOS1-controlled renin on blood pressure changes. Lack of macula densa NOS1 inhibited renin release and delayed the recovery of blood pressure following hemorrhagic shock.

The macula densa is the tubular component of juxtaglomerular apparatus (JGA) located at the distal segment of the thick ascending limb (TAL). Macula densa cells are specialized epitheliums and serve as a sensor of the alter in luminal NaCl concentrations. An increase in tubular NaCl concentration activates Na-K-2Cl cotransporters (NKCC2) at the macula densa, which enhances vascular tone and constricts the afferent arterioles by a mechanism of tubuloglomerular feedback (TGF) [2,19]. Increases in tubular sodium concentration and flow rate also induce NO generation by the macula densa nitric oxide synthase 1 (NOS1), which in turn, blunts TGF response [20]. In addition, macula densa NO also plays a unique role in the regulation of renin release [2, 3, 21, 22].

By crossing NKCC2-Cre mice with NOS1flox/flox mice, all three splice variants of NOS1 were exclusively deleted from the macula densa. In our previous report, we confirmed the functional deletion of NOS1 in the MDNOS1KO mice by demonstrating a significant reduction in NO generation by the macula densa compared to the WT [9]. This mouse line provides a unique tool to dissect the significant role of macula densa NOS1 in the control of renin release *in vivo*. The similar GFR values observed in WT and MDNOS1KO mice fed different salt diets demonstrate that the specific deletion of the *NOS1* gene in the macula densa did not have an impact on kidney function. The results that a low salt diet stimulates PRC, while a high salt diet suppresses PRC are consistent with the previous reports [23, 24, 25]. A restriction in sodium intake markedly increased the PRC and a high salt intake reduced PRC in WT mice in this study. However, the increase in PRC stimulated by a low salt diet was significantly dulled in the MDNOS1KO mice, indicating the fundamental function of macula densa NO in the stimulation of renin release. Additionally, the suppressed PRC in the MDNOS1KO mice with high salt diet further confirmed the significant role of macula densa NO in the mediation of renin release. Hypertension in the MDNOS1KO induced by a high salt diet was reported in our previous study [9], while the increased blood pressure with high salt diets in the MDNOS1KO mice appears to be independent of PRC.

In isolated perfused JGA, NOS inhibition was found to block the renin release in response to a low tubular sodium concentration [26, 27, 28]. An acute infusion of NOS1 inhibitor, 7-nitroindazole (7-NI), blocked the renin release induced by a bolus injection of furosemide, an acute stimulator of renin release [29, 30]. While the conclusions of these studies are in agreement with our findings, inconsistent observations exist. Some studies reported that changes in salt intake induced similar PRC between global NOS1 KO and WT mice [31]. Non-selective NOS inhibition with L-NG-Nitroarginine methyl ester or selective NOS1 inhibition with 7-NI did not show any effects on renin release in response to changes in salt intake [6]. Several factors may contribute to the discrepancy in the results. First, in the global NOS1 KO mice used in previous studies, the gene modification was targeted at exon 1 and only the NOS1α isoform was deleted. The expression of NOS1β was intact in these mice [9]. NOS1β is the major splice variant and accounts for most of the NO generation by the macula densa. Therefore, macula densa NO generation is largely unchanged in the global NOS1 KO mice [9, 32]. Second, NOS inhibition raises blood pressure, which also affects renin release [22, 33, 34].

Renal perfusion pressure (RPP) is an essential factor in control of renin release, mediated by baroreceptor reflex and sympathetic activity [35, 36, 37], as well as activity of RAAS [21]. To further determine the role of macula densa NOS1 in renin release, and more importantly, to examine the functional significance of the macula densa NOS1-mediated renin on blood pressure, we induced hemorrhagic shock by withdrawing 0.4 mL of blood in WT and MDNOS1KO mice and compared the PRC and MAP levels. Hemorrhagic shock led to about a 3-fold increase of PRC in WT mice, while only about a 54% elevation of PRC in MDNOS1KO mice. In addition, the MAP was significantly lower during 0.5–6 hours after hemorrhagic shock with a slower recovery in the MDNOS1KO mice compared with the WT mice. This data, for the first time, demonstrated the functional significance of macula densa NOS1 in the control of blood pressure, which is possibly mediated by the control of PRC after hemorrhagic shock. Our data also suggested that the renin secretory response after bleeding occurred very rapidly [38, 39, 41], since the differences in MAP were observed within 10 minutes and reached significant levels at the nadirs within 30 min after hemorrhagic shock. MAP returned to baseline within 12 hours after hemorrhagic shock, indicating the powerful recovery mechanisms including RAAS activation. Other mechanisms underlying macula densa NOS1 regulated renin release are to be determined in the future studies, which may occur via the NO signaling pathway [2, 3, 33] by modulation of TGF response that alters vascular tone of the afferent arterioles, or sympathetic activities [9]. GFR in conscious mice provides supporting data for the different dietary intakes. A low salt diet reduces GFR, while a high salt diet elevates GFR. However, there are no significant differences between the WT and KO mice fed a normal, low, or high salt diet, [9] suggesting compensatory responses.

The first line response following hemorrhagic shock is the activation of the sympathetic system, which almost instantly increases vessel constriction, enhances tubular sodium transporter activity, and stimulates RAS activity. However, since the heart rates, a marker of sympathetic activity, had no significant differences between the MDNOS1KO and WT mice, it is unlikely that the deletion of macula densa NOS1 significantly affects sympathetic activity, nor that the changes in MAP observed in our study are due to differences in sympathetic activity.

## Conclusions

In conclusion, NO derived from macula densa-NOS1 acts as an essential factor in mediating the renin release and in maintaining blood pressure in hypovolemic situations. Targeting macula densa NOS1 may be a novel strategy in the blood pressure management by regulation of renin release.

## Supplementary Material

Supplementary Material

## Data Availability

All the data and materials supporting the findings of this study are available within the article. The raw data supporting the conclusion of this article will be made available by the authors.

## Abbreviations

RAAS: Renin-angiotensin-aldosterone system
TAL: thick ascending limb
JGA: juxtaglomerular apparatus
NO: nitric oxide
TGF: tubuloglomerular feedback
FITC-sinistrin: fluorescein isothiocyanate-sinistrin
MDNOS1KO: Macula densa-specific NOS1 knockout
PRC: Plasma renin concentration
MAP: Mean arterial pressure
PAC: Plasma aldosterone concentration
NOS1: nitric oxide synthase 1
GFR: glomerular filtration rate
