# Supplementary material for: Macula Densa Nitric Oxide Synthase-1 Controls Renin Release and Renin-Dependent Blood Pressure Changes
Source: Discov Med. Author manuscript; Available in PMC 2024 Mar 8. (PMC10921921; doi:10.24976/Discov.Med.202335177.53)
Supplement: Supplementary Material [file NIHMS1967909-supplement-Supplementary_Material.docx]

Supplementary Materials

# Immunofluorescence staining

Paraffin embedded Kidney samples for both WT and MDNOS1KO mice were sectioned into 4-μm slices. Immunofluorescence staining of the kidney slices with the rabbit anti-Renin primary antibody (Abcam, AB212197, Waltham, MA, USA) and a fluorophore-conjugated secondary antibody Donkey Anti-Rabbit IgG H&L (Abcam, ab150075, Waltham, MA, USA) was completed as described previously [1,2]. The tissue sections were photographed with a fluorescence microscope (Keyence BZ-X710, Itasca, IL, USA) and analyzed with Fiji/ImageJ. Five images per kidney were acquired and the relative renin density was calculated by dividing the renin-positively stained area by the kidney area. The morphometric analyses were conducted in a blind manner with respect to the experimental procedures.

# Renin mRNA expression measurement by Real-time PCR

To evaluate the impact of MD-specific NOS1 deletion on intrarenal RAS, we utilized Real-time PCR to measure the mRNA expression of renin, following the established methodology described earlier [3,4]. β-actin served as the reference gene for internal standardization purposes. Relative quantitative expression of renin was determined using SYBR Green I (Invitrogen Molecular Probes, Eugene, OR, USA) in a CFX Connect system (BioRad, Hercules, CA, USA). Ren1 mRNA (primer sequence: forward, 5’-ACAGTATCCCAACAGGAGAGACAAG-3’, reverse, 5’-GCACCCAGGACCCAGACA-3’) expression was adjusted with β-actin expression, and the relative changes in expression were calculated using the ∆∆Ct method and expressed as relative expression in comparison to WT mice.


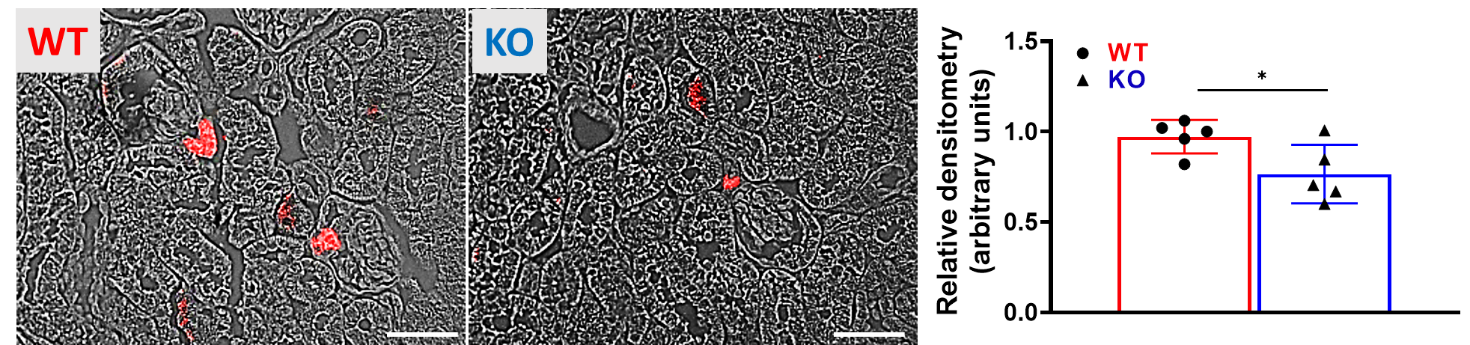


**Supplementary Fig. 1.** **Immunofluorescence staining was performed as described in the methods section [40, 42].** Representative digital images from kidneys of wild type and MDNOS1KO mice taken at 400× magnifications (scale bar = 50 µm), showed specific renin staining (red) in the juxtaglomerular (JG) cells. Quantification of red staining in wild type and MDNOS1KO mice showed less renin positive cells in the MDNOS1KO mice than in the WT mice (**p* < 0.03, MDNOS1KO vs. WT; n = 5).

**Supplementary Fig. 2. Renin mRNA expression in WT and MDNOSKO mice under different salt diets.** Relative to control NS, renin mRNA expression in the WT mice has been stimulated by salt restriction and suppressed by high salt intake (****p* < 0.001, MDNOS1KO vs. WT at LS; n = 5). However, the renin mRNA expression in the MDNOSKO mice did not show a clear response to the salt stimulation.

References

[1] Chen W, Wang L, Liang P, Mast J, Mathis C, Liu CY, *et al*. Reducing ischemic kidney injury through application of a synchronization modulation electric field to maintain Na^+^/K^+^-ATPase functions. Science Translational Medicine. 2022; 14: eabj4906.

[2] Wei J, Zhang J, Wang L, Jiang S, Fu L, Buggs J, *et al*. New mouse model of chronic kidney disease transitioned from ischemic acute kidney injury. American Journal of Physiology. Renal Physiology. 2019; 317: F286–F295.

[4] Wang L, Song J, Wang S, Buggs J, Chen R, Zhang J, *et al*. Cross-sex transplantation alters gene expression and enhances inflammatory response in the transplanted kidneys. American Journal of Physiology. Renal Physiology. 2017; 313: F326–F338.
